# Revisiting the Gray Zone in HBeAg-Negative Chronic Hepatitis B: Insights From a Multicenter Liver Biopsy Cohort

**DOI:** 10.5152/tjg.2026.26367

**Published:** 2026-08-11

**Authors:** Özlem Gül, Hüseyin Köseoğlu, Selim Yalçın, Barış Yılmaz, Dilek Oğuz, Sema Zergeroğlu, Bilal Ergül

**Affiliations:** 1 Department of Gastroenterology, Lokman Hekim University Faculty of Medicine, Ankara, Türkiye; 2 Department of Gastroenterology, Hitit University Faculty of Medicine, Çorum, Türkiye; 3 Department of Oncology, Kırıkkale University Faculty of Medicine, Kırıkkale, Türkiye; 4 Department of Gastroenterology, Kırıkkale University Faculty of Medicine, Kırıkkale, Türkiye; 5 Department of Pathology, Kırıkkale University Faculty of Medicine, Kırıkkale, Türkiye

**Keywords:** Chronic hepatitis B, gray zone, HBeAg-negative chronic hepatitis B, liver biopsy, normal ALT, significant fibrosis

## Abstract

**Background/Aims:**

The management of patients with HBeAg-negative chronic hepatitis B (CHB) with intermediate viremia and persistently normal alanine aminotransferase (ALT) levels remains controversial. This “gray-zone” (GZ) population may harbor significant histological liver injury despite not meeting conventional treatment criteria. This study characterized the histologic features of this GZ population and compared their clinical and laboratory characteristics.

**Materials and Methods:**

In this multicenter retrospective study, patients with HBeAg-negative CHB who had hepatitis B virus (HBV) DNA levels between 2000 and 20 000 IU/mL and persistently normal ALT levels and who underwent liver biopsy between 2014 and 2022 were included. Histological assessment was performed using the modified Ishak scoring system. Significant fibrosis was defined as Ishak stage of 2 or greater. Clinical, virological, and noninvasive fibrosis parameters were analyzed.

**Results:**

A total of 145 patients were included (mean age: 46.3 ± 12.6 years; 54.5% female). Significant fibrosis and/or moderate-to-severe necroinflammatory activity requiring antiviral therapy was identified in 109 patients (75.1%). Although ALT and aspartate aminotransferase levels remained within normal reference range, both were significantly higher in the treatment group compared than in the nontreatment group (*P* = .003 and *P* = .018, respectively). Aspartate aminotransferase-to-platelet ration index (APRI) scores were also significantly elevated among treated patients (*P* = .02), whereas Fibrorsis-4 (FIB-4) scores did not differ significantly. HBV DNA and quantitative Hepatitis B surface antigen (HBsAg) levels were comparable between groups.

**Conclusion:**

A substantial proportion of patients with HBeAg-negative CHB in the GZ exhibited clinically significant histologic disease despite persistently normal ALT levels. Reliance solely on conventional biochemical thresholds may underestimate the burden of fibrosis. Early histological evaluation and more proactive treatment strategies should be considered for selected patients in the GZ.

**Main Points**

- Significant fibrosis and/or necroinflammatory activity was common among patients with HBeAg-negative chronic hepatitis B in the gray zone despite persistently normal alanine aminotransferase (ALT) levels.
- Three out of 4 patients in this cohort met histologic criteria for antiviral treatment.
- Higher ALT, aspartate aminotransferase, and APRI values within the normal reference range were associated with eligibility for treatment, whereas FIB-4, HBV DNA, and HBsAg levels were not.
- Liver biopsy remains valuable for risk stratification in patients with inconclusive biochemical findings.

## Introduction

Chronic hepatitis B (CHB) remains a major cause of liver-related morbidity and mortality worldwide despite the widespread implementation of vaccination programs and the availability of highly effective antiviral therapies. According to recent estimates, approximately 254 million people are living with chronic hepatitis B virus (HBV) infection worldwide, and nearly 2 billion individuals have been exposed to HBV. Persistent HBV infection may lead to progressive hepatic fibrosis, cirrhosis, hepatic decompensation, and hepatocellular carcinoma (HCC), making CHB a continuing global health concern.^1^^,^^2^

The introduction of potent nucleos(t)ide analogue (NA) therapy has substantially altered the natural course of CHB. Sustained viral suppression has been associated with reduced hepatic inflammation and fibrosis progression, regression of early cirrhotic changes, and a lower incidence of HCC. Furthermore, long-term antiviral treatment has been shown to improve liver-related outcomes and overall survival in appropriately selected patients.^3^^,^^4^

Current treatment recommendations from major international societies, including the American Association for the Study of Liver Diseases (AASLD), the European Association for the Study of the Liver (EASL), and the Asian Pacific Association for the Study of the Liver (APASL), primarily rely on a combination of serum alanine aminotransferase (ALT) levels, HBV DNA concentrations, and fibrosis severity to determine treatment eligibility.^2^^,^^5^^,^^6^ As a result, patients who do not meet these predefined criteria are generally monitored without antiviral intervention.

However, a considerable proportion of individuals with chronic HBV infection do not fit neatly into the traditional phases of disease. Many of these patients exhibit intermediate levels of viral replication together with persistently normal or minimally elevated ALT values and are frequently categorized as belonging to the so-called “gray zone” (GZ). Increasing evidence suggests that this group represents a clinically heterogeneous population in whom significant necroinflammatory activity and fibrosis may be present despite apparently reassuring biochemical findings. Consequently, the ability of ALT alone to accurately reflect underlying liver injury has increasingly been questioned.

Management of GZ patients remains challenging because current guidelines provide limited guidance regarding the optimal timing of treatment initiation. Advances in noninvasive fibrosis assessment, including transient elastography (TE) and magnetic resonance elastography, as well as emerging virological biomarkers such as quantitative HBsAg, HBV RNA, and hepatitis B core–related antigen (HBcrAg), have improved disease stratification. In addition, novel predictive models and artificial intelligence–assisted approaches are being explored to facilitate individualized clinical decision-making.^2^^,^^5^^,^^6^ These developments have renewed interest in identifying patients who may benefit from earlier therapeutic intervention before irreversible liver injury occurs.

Despite growing attention to this population, the histopathological burden of disease among patients with HBeAg-negative CHB who had intermediate HBV DNA levels and persistently normal ALT levels remains incompletely defined. Because liver biopsy is not routinely performed in most patients in the GZ, clinically significant fibrosis may remain undetected, and treatment initiation may be unnecessarily delayed. Therefore, this multicenter study aimed to evaluate the histologic characteristics of patients with HBeAg-negative CHB who had HBV DNA levels between 2000 and 20 000 IU/mL and persistently normal ALT levels who underwent liver biopsy. The authors also sought to determine clinical and laboratory factors associated with significant fibrosis in this challenging subgroup.

## Materials and Methods

### Study Design

This multicenter retrospective cohort study was conducted at the Gastroenterology Clinics of Kırıkkale University Faculty of Medicine, Hitit University Faculty of Medicine, Ahi Evran University Training and Research Hospital, and Çankırı State Hospital. Medical records of patients who underwent liver biopsy for CHB between January 2014 and May 2022 were systematically reviewed.

### Study Population

Although recent literature stratifies the heterogeneous indeterminate (GZ) population into 4 distinct subgroups (groups A through D), this study specifically focused on patients in group C, defined as being HBeAg-negative with ALT levels below the upper limit of normal (ALT < ULN) and an HBV DNA viral level of ≥ 2000 IU/mL. Other GZ subcategories (groups A, B, and D) were excluded to maintain cohort homogeneity and specifically investigate the risk of silent fibrotic progression unique to group C individuals.

**Inclusion Criteria**

- Age ≥18 years
- HBeAg-negative CHB infection (HBsAg positivity ≥6 months)
- At least 2 HBV DNA measurements between 2000 and 20 000 IU/mL within the 12 months preceding liver biopsy
- Persistently normal ALT levels, defined as 4 consecutive ALT values <40 IU/L within a 12-month period

**Exclusion Criteria**

- Prior antiviral therapy
- Hepatitis D virus infection
- Hepatitis C virus coinfection
- HIV coinfection
- Significant alcohol consumption (>20 g/day for women, >30 g/day for men)
- Hepatic steatosis >5% on liver biopsy (indicative of concomitant metabolically related liver disease/MASLD)
- Cirrhosis or HCC
- Wilson’s disease
- Hereditary hemochromatosis
- Autoimmune hepatitis
- Cholestatic liver diseases
- Ongoing immunosuppressive therapy
- Pregnancy

### Liver Biopsy Procedure and Histopathological Evaluation

Percutaneous liver biopsies were performed under ultrasound guidance by experienced gastroenterologists using 18-gauge Menghini needles. Only specimens measuring >20 mm in length and containing ≥6 portal tracts were considered adequate for histopathologic evaluation.

Specimens were fixed in formalin, dehydrated, paraffin embedded, sectioned into micrometer-thick slices, and stained with hematoxylin and eosin. Histological assessment was performed using light microscopy.

Fibrosis stage and necroinflammatory activity were graded according to the modified Histology Activity Index (Ishak scoring system). Fibrosis was staged on a 7-point scale (0-6), and necroinflammatory activity was scored on an 18-point scale.

Significant fibrosis was defined as Ishak stage ≥2. Moderate-to-severe necroinflammatory activity was defined as Histology Activity Index (HAI) score ≥6.

All biopsy specimens were independently reviewed by an experienced hepatopathologist. Discordant findings, if present, were resolved by consensus.

Antiviral therapy was initiated in patients with fibrosis stage ≥2 and/or HAI score ≥6, in accordance with institutional clinical practice.

### Clinical and Laboratory Assessment

Relevant demographic, biochemical, and virologic data obtained at the time of liver biopsy were retrospectively collected from patient records. The analyzed parameters included serum ALT and aspartate aminotransferase (AST), which reflect hepatic inflammatory activity, as well as hemoglobin, platelet count, quantitative HBsAg, and HBV DNA levels.

Serum ALT and AST levels were measured using an automated biochemical analyzer (e.g., Roche Cobas 8000, Roche Diagnostics, Mannheim, Germany) based on standardized enzymatic colorimetric assays. The upper limit of normal (ULN) for ALT was defined as 40 IU/L according to the institutional laboratory reference range.

Complete blood count parameters, including hemoglobin and platelet count, were measured using an automated hematology analyzer (e.g., Sysmex XN-1000, Sysmex Corporation, Kobe, Japan).

Quantitative HBsAg levels were measured using a chemiluminescent microparticle immunoassay (e.g., Architect HBsAg QT assay; Abbott Diagnostics, Abbott Park, IL, USA) and were expressed in IU/mL according to World Health Organization (WHO) standards.

Serum HBV DNA levels were quantified using a real-time polymerase chain reaction assay (e.g., COBAS AmpliPrep/COBAS TaqMan HBV Test v2.0, Roche Diagnostics). The lower limit of detection was 20 IU/mL, and results were expressed in IU/mL.

All laboratory measurements were performed in certified clinical laboratories of the participating centers, according to the manufacturer’s instructions and standardized quality control procedures.

### Noninvasive Fibrosis Indices

Noninvasive fibrosis indices were calculated using laboratory parameters obtained on the day of liver biopsy:

APRI = [(AST / ULN of AST)/platelet count (10⁹/L)] × 100

FIB-4 = [Age × AST (IU/L)]/[platelet count (10⁹/L) × √ALT (IU/L)]

### Outcome Measures

The primary outcome was the presence of significant fibrosis (Ishak ≥2) among GZ patients.

Secondary outcomes included:

- Moderate-to-severe necroinflammatory activity (HAI score ≥6)
- Initiation of antiviral therapy based on histological findings

### Statistical Analysis

Statistical analyses were performed using SPSS version 30.0 (IBM SPSS Corp.; Armonk, NY, USA).

Continuous variables are expressed as mean ± SD or median (interquartile range) according to distribution. Categorical variables are presented as number and percentage.

Data distribution was assessed using the Kolmogorov–Smirnov test and visual inspection of histograms. Depending on the distribution of the variables, comparisons between groups were performed using either the independent-samples *t* test or the Mann–Whitney *U*-test for continuous variables. Categorical variables were analyzed using the chi-square test or Fisher’s exact test, as appropriate.

### Sample Size and Power Analysis

A post-hoc power analysis was performed based on the observed prevalence of significant fibrosis. With a total sample size of 145 patients and a 2-sided alpha of 0.05, the study achieved >80% power to detect moderate effect sizes in fibrosis risk between subgroups.

All statistical tests were 2-tailed, and *P* < .05 was considered statistically significant.

**Ethical Considerations**

Ethical committee approval was obtained from the Ethics Committee of University of Kırıkkale (Approval no: 2025.09.02, Date: September 24, 2025). The study was conducted in accordance with the Declaration of Helsinki. Given the retrospective design, the requirement for informed consent was waived.

ChatGPT (OpenAI, San Francisco, CA, USA) was used for language editing during manuscript preparation. However, all scientific content, interpretations, and conclusions were reviewed and approved by the authors.

## Results

A total of 145 patients were included in the study, with a mean age of 46.36 ± 12.6 years. The cohort demonstrated a slight female predominance (54.5%, n = 79). The mean body mass index (BMI) was 26.37 ± 3,7 kg/m². Baseline biochemical parameters showed mean ALT and AST levels of 24.48 ± 8.1 U/L and 22.24 ± 5.91 U/L, respectively, both within generally accepted normal reference ranges. The mean hemoglobin level was 14.33 ± 1.58 g/dL, and the mean platelet count was 234.33 ± 55.59 ×10⁹/L. Virologic assessment showed mean HBsAg and HBV DNA levels of 6423.52 ± 2245.64 IU/mL and 7784.91 ± 5316.8 IU/mL, respectively (Table 1).

Patients were stratified into treatment (n = 109) and nontreatment (n = 36) groups. The 2 groups were comparable in terms of baseline demographic characteristics, with no significant differences observed in age, age distribution, BMI, hemoglobin levels, or platelet counts (*P* > .05 for all) (Table 2).

Virological parameters were similarly distributed between the groups. Mean HBsAg and HBV DNA levels did not differ significantly between the treatment and nontreatment groups (*P* = .12 and *P* = .16, respectively), indicating comparable baseline viral burden.

Despite transaminase levels remaining within normal laboratory limits in both groups, statistically significant differences were observed. Patients in the treatment group had higher ALT levels than those in the nontreatment group (25.63 ± 7.81 vs. 21.02 ± 8.07 U/L, *P* = .003). Similarly, AST levels were significantly higher in the treatment group (22.90 ± 5.94 vs. 20.22 ± 5.43 U/L, *P* = .018).

Assessment of noninvasive fibrosis markers revealed a significantly higher APRI score in the treatment group (0.26 ± 0.11 vs. 0.21 ± 0.06, *P* = .02). Although FIB-4 scores were numerically higher in treated patients, this difference did not reach statistical significance (1.01 ± 0.56 vs. 0.85 ± 0.37, *P* = .11).

## Discussion

The clinical management of patients in the GZ of CHB has emerged as one of the most debated topics in hepatology over the past decade. These patients, often categorized as being in an “indeterminate phase,” do not meet the classical criteria for treatment initiation defined by major international guidelines.^7^^-^^9^ However, recent longitudinal data and histological studies suggest that the GZ is far from a stable or “inactive” state, frequently harboring significant liver injury and a high risk of long-term complications.^10^^,^^11^

The histological findings outlined in Table 2 demonstrate that patients in the treatment group had significantly higher baseline HAI scores and advanced Ishak fibrosis stages than those in the untreated group. This clear pathological divergence underscores the critical role of liver biopsy in unmasking significant subclinical necroinflammation and early-stage fibrosis within the GZ type C population, thereby providing objective guidance for antiviral therapy initiation.

Recent large-scale studies indicate that a substantial proportion of the CHB population ranging from 27.8% to 38.7% falls within the GZ.^7^^,^^12^ The study finding that 75.1% of HBeAg-negative GZ patients had significant histological disease is strongly supported by the literature from the past 10 years. For example, Wang et al^13^ reported that more than 70% of GZ patients had significant histological disease, whereas other cohorts have shown that up to 54.2% of patients in the indeterminate phase have moderate-to-severe inflammation. Even among patients with persistently normal ALT levels, significant liver histologic changes are observed in approximately 72.4% of cases.^14^ These findings challenge the traditional wait-and-see approach and suggest that viral replication (HBV DNA >2000 IU/mL), even in the presence of normal ALT levels, may contribute to silent disease progression.^10^

A critical insight from the past decade is the unreliability of traditional ALT thresholds (typically 40 U/L) for identifying at-risk GZ patients. The study observed significant histological damage even when ALT levels were within laboratory reference ranges. Data from 2021 suggest that nearly 30.8% of patients with “normal” ALT harbor severe inflammation or advanced fibrosis.^15^ This has prompted a global shift toward lower ALT thresholds; for example, recent guidelines have recommended thresholds as low as 30 U/L for men and 19 U/L for women to identify at-risk patients earlier.^16^ Achieving early ALT normalization is now recognized as an important factor in reducing the cumulative incidence of HCC.^17^

The significance of the GZ is most apparent when considering long-term prognosis. Recently published 10-year follow-up data revealed that patients remaining in the indeterminate/GZ phase had a 14-fold higher risk of developing HCC than those in a truly inactive phase.^7^ The 10-year cumulative incidence of HCC was significantly higher among GZ patients (4.6%) than among inactive carriers (0.5%).^7^ Furthermore, GZ patients have higher rates of disease progression and end-stage liver events than inactive CHB groups.^11^ This elevated risk highlights that the GZ may represent a transitional state toward active disease rather than a benign phase.^7^

The benefits of initiating antiviral therapy in GZ patients have become clearer with recent evidence. Antiviral treatment in this population is associated with a 70% reduction in HCC risk.^18^ In longitudinal studies, therapy significantly reduced fibrosis progression among patients with elevated DNA levels and near-normal ALT levels.^7^ Beyond individual outcomes, expanding treatment to GZ patients (HBV DNA ≥ 2000 IU/mL regardless of ALT) is increasingly recommended to simplify management and reduce community transmission.^10^^,^^19^

Although noninvasive tests such as APRI and FIB-4 are valuable screening tools, their performance in the GZ remains a subject of scrutiny.^20^ The results showed significantly higher APRI scores in the treatment-eligible group, consistent with data demonstrating that these markers have high negative predictive values for excluding cirrhosis.^21^ However, noninvasive tests often lack the sensitivity to distinguish mild from moderate fibrosis in GZ patients, for whom liver biopsy remains the reference standard.^22^^,^^23^ Current guidelines emphasize that when biochemical markers are in the GZ, histological evaluation or advanced imaging is critical to avoid withholding necessary therapy.^8^^,^^9^^,^^24^

Although liver biopsy remains the gold standard diagnostic tool in this study, its routine application in clinical practice is often limited by its invasiveness and the risk of complications. To overcome these limitations, noninvasive fibrosis assessment modalities have become increasingly utilized. Among these, TE is widely recognized as a reliable tool for excluding advanced fibrosis and cirrhosis in CHB.^25^^,26^ Nonetheless, in the GZ population, TE may yield equivocal or borderline results because of ongoing subclinical liver inflammation or the absence of established cutoff values for this unique population.

A limitation of this study is the use of a conventional ALT ULN threshold of 40 IU/L. The definition of a “normal“ ALT level remains a subject of ongoing debate among major societies. Although the AASLD proposes lower, sex-specific ULN thresholds (35 U/L for men and 25 U/L for women), the APASL guidelines explicitly define the ULN as 40 IU/L, and EASL guidelines also align more closer with this conventional threshold in clinical practice.^8^^,^^9^^,^^27^ Because a substantial proportion of the cohort had ALT values clustered around 20-25 IU/L, applying the stricter AASLD criteria might reclassify some patients from the GZ to the abnormal category. Although the use of 40 IU/L reflects real-world laboratory practices and is consistent with APASL/EASL benchmarks, future prospective studies comparing these differing threshold systems are warranted.

In conclusion, evidence from the past decade indicates that the GZ represents a high-risk population characterized by significant histologic activity and a substantial risk of HCC.^7^^,^^13^ These findings, together with the recent literature, support a more proactive management strategy. Rather than passive monitoring, clinicians should consider early biopsy or the initiation of antiviral therapy to reduce the risk of irreversible liver damage and malignancy in GZ patients.

## Figures and Tables

**Table 1.** .Baseline Demographic and Laboratory Characteristics of the Study Population

Variable	Overall Cohort (N = 145)
Age, years	46.36 ± 12.6
Gender, n (%)	
Female	79 (54.5)
Male	66 (45.5)
BMI, kg/m²	26.37 ± 3.7
ALT, IU/L	24.48 ± 8.1
AST, IU/L	22.24 ± 5.91
Hemoglobin, g/dL	14.33 ± 1.58
Platelet count, ×10⁹/L	234.33 ± 55.59
HBsAg, IU/mL	6423.52 ± 2245.64
HBV DNA, IU/mL	7784.91 ± 5316.8

Data are presented as mean ± SD or number (%).

ALT, alanine aminotransferase; AST, aspartate aminotransferase; BMI, body mass index; HBV, hepatitis B virus.

**Table 2.** Comparison of Baseline Demographic and Laboratory Characteristics Between Treatment and Nontreatment Groups

Variable	Treatment Group (n = 109)	Nontreatment Group (n = 36)	*P*
Age, years	47.33 ± 13.06	43.41 ± 10.74	.10
Age ≥40 years, n (%)	77	24	.65^†^
Age <40 years, n (%)	32	12	
BMI, kg/m²	26.48 ± 3.69	24.82 ± 4.25	.45
ALT, U/L	25.63 ± 7.81	21.02 ± 8.07	**.003**
AST, U/L	22.90 ± 5.94	20.22 ± 5.43	**.018**
Hemoglobin, g/dL	14.39 ± 1.52	14.15 ± 1.78	.43
Platelet count, ×10⁹/L	230.65 ± 53.66	245.47 ± 60.47	.16
HBsAg, IU/mL	6588.30 ± 2262.62	5924.61 ± 2147.13	.12
HBV DNA, IU/mL	8140.27 ± 5549.00	6708.94 ± 4438.57	.16
HAI	6.21 ± 1.48	3.38 ± 0.80	**.001**
Fibrosis score	2.09 ± 0.78	0.66 ± 0.47	**.001**
APRI score	0.26 ± 0.11	0.21 ± 0.06	**.02**
FIB-4 score	1.01 ± 0.56	0.85 ± 0.37	.11

Data are presented as mean ± SD or number (%).

Statistically significant P values (P < .05) are shown in bold.

ALT, alanine aminotransferase; AST, aspartate aminotransferase; BMI, body mass index.

† Chi-square test applied for categorical comparison.

## Data Availability

The data that support the findings of this study are available on request from the corresponding author.
